# Readability levels and thematic content analysis of online and printed lead poisoning informational materials

**DOI:** 10.1186/s12889-021-11944-w

**Published:** 2021-10-17

**Authors:** Harriet Okatch, Ebony Pitts, Emily Ritchey, Kylie Givler, Madeline Kuon

**Affiliations:** 1 Biology Department, Franklin and Marshall College, 415 Harrisburg Avenue, Lancaster, PA 17603 USA; 2 Department of Physical Therapy, University of Delaware, 210 South College Ave, Newark, DE 19716 USA

**Keywords:** Lead poisoning, Readability levels, Informational materials, Thematic content analysis, Health communication

## Abstract

**Background:**

Lead poisoning prevention efforts include preparing and disseminating informational materials such as brochures and pamphlets to increase awareness of lead poisoning, lead exposures and lead poisoning prevention. However, studies have demonstrated that patient education materials for diseases and health conditions are prepared at a reading level that is higher than the recommended 7th–8th grade reading level. This study, therefore, aims to assess the reading levels of lead poisoning informational materials.

**Methods:**

Lead poisoning materials (*N* = 31) were accessed from three states; Michigan, New York and Pennsylvania. The readability levels of the materials were assessed using the Flesh Kincaid Grade Level readability test. The Kruskal-Wallis test was conducted to determine if the readability levels differed between the materials obtained from the different states. Thematic content analyses were carried out to assess the inclusion of four themes; definition of lead poisoning, risk factors and exposures, testing and referral and prevention covering 12 subtopics. The Wilcoxon rank sum test was used to examine if there was a difference in the number of subtopics by readability level (dichotomized to >8th grade and < 8th grade).

**Results:**

The median readability level of the informational materials was 6.7 (IQR: 5.1–8.1). However, there was variability in the readability levels of the materials (range 3.5 to 10.6); materials obtained from Michigan had the highest median reading level of 8.1 (IQR: 6.9–9.0) followed by Pennsylvania. Heterogeneity was observed in the content of the materials. Most of the materials (80%) from Michigan focused on water as a source of lead poisoning, whereas materials from New York and Pennsylvania focused on lead-based paint and other sources. The materials prepared at >8th grade reading level contained fewer topics than materials prepared at <8th grade reading level.

**Conclusions:**

We find that the materials were often prepared at reading levels lower than the recommended 8th grade reading level. However, there is variability in the reading levels and in the content of the materials. While the materials met the general readability guidelines, they did not necessarily meet the needs of specific groups, especially groups at risk.

## Background

Health communication defined by the Centers for Disease Control and Prevention (CDC) as “the study and use of communication strategies to inform and influence individual decisions that enhance health” [1] includes both verbal and written media to promote the health of individuals and communities. Pamphlets and brochures are a subset of general health communication material that can provide individuals with information about specific health related topics. The aims of these health promotion materials are to increase knowledge, and promote understanding thereby allowing individuals to assess perceived risks, to develop self-efficacy to promote positive behaviors, and/or to direct individuals to additional relevant resources. For health communication to be effective, the design, packaging and framing of the message should consider audience/consumer characteristics including literacy and language.

Despite the availability of several educational materials addressing lead poisoning, related exposures, effects and associated preventive measures, childhood lead poisoning continues to affect, nationally, 3.0% of children under the age of 6 years [2]. Children with elevated blood levels are at risk of adverse neurodevelopmental effects [3], lower IQ scores and behavioral issues [4–6]. In addition, several studies, both qualitative and quantitative, have demonstrated that knowledge levels about lead and lead poisoning are low [7–10]. Because readable texts are effective instruments to increase awareness, transfer knowledge and initiate behavior change with respect to a specific health event [11, 12], it is important to understand why lead poisoning continues to persist given the magnitude of educational materials that have been developed.

Several studies have demonstrated that the patient education materials (PEM) for diseases and health conditions are prepared at a reading level that is higher than the 7th–8th grade reading level [13–17]. The Centers for Disease Control and Prevention (CDC), other health agencies, and researchers recommend or demonstrate health related material be prepared at a 5th to 8th grade reading level for effective comprehension by the audience and yet reading levels for health-related material are usually prepared at higher reading levels [18–22]. Readability is defined as “the ease with which written materials are read” [23].

We hypothesize that the readability levels of lead poisoning prevention materials are not tailored to the guidelines of the CDC, and therefore are inadequate in their task of educating the American public on the realities of lead poisoning. Therefore, we aim to assess the readability levels of lead poisoning informational/educational materials. In addition, we will assess the thematic content of the materials to identify the salient topics included in the informational materials.

## Methods

A total of 31 brochures on lead and lead poisoning were accessed from three states; Michigan (MI), New York (NY) and Pennsylvania (PA). Twenty-four of the materials were accessed online from webpages of local, and state health authorities. 5 of the materials were physical brochures/pamphlets received from the City of Lancaster in Pennsylvania (PA), 1 brochure was obtained from the Partnership for Public Health, a non-profit organization in Lancaster, PA, and 1 door hanger was obtained from Catholic Health Initiative (CHI) St Joseph’s Children’s Health, a hospital providing pediatric care in Lancaster, PA. All the other PA materials were from federal agencies including the CDC and the Environmental Protection Agency.

Specifically, Michigan (MI) was selected because of the recent water crisis and the associated lead poisoning cases in Flint which rekindled national conversations around lead poisoning in 2014 [24]; New York was selected because of the numerous lead laws, ordinances and policies [25] that have been passed because of the relatively high prevalence of childhood lead poisoning (14. 7 per 1000 tested children younger than 6 years of age [26]); and Pennsylvania was selected because the City of Lancaster where the researchers conduct lead poisoning prevention research is in the state of Pennsylvania; Pennsylvania was recorded as having one of the highest prevalence of childhood lead poisoning in the country at 5.1% [2].

### Readability levels

All materials were produced in English. The materials were reformatted in Microsoft Word, all figures, hyperlinks, acknowledgements and references were removed, and punctuation marks were added where applicable. The Flesch Kincaid Grade Level (FKGL) readability test was used to determine the readability level for each resource [27]. The output of the FKGL is an integer between 0 and 18. The integers denote that individuals who have completed the corresponding grade level should be able to read the text; for example, a resource that yields an output of 10 suggests that individuals who have completed the 10th grade in the United States of America should be able to read and understand the material presented in the resource. The interpretation and the relative ease of reading are presented in Table 1.

**Table 1:** The Flesch Kincaid Grade Level formula and the interpretation of the test

	Formula	Interpretation
FKGL	0.39 (total words/total sentences) + 11.8 (total syllables/total words) – 15.59	5: Very easy to read (5th grade) 6: Easy (6th grade) 8: Standard (8th grade) 10: Fairly difficult (10-12th grade) 12–14: Difficult (College level) > 14: Very difficult (College graduates)

### Thematic content analyses

All content was thematically analyzed for the following topics (4 themes and 12 subtopics); i) definition of lead poisoning (symptoms and effects), ii) Risk factors and exposures (old housing, lead-based paint/lead dust, water, soil, food, pottery, cosmetics), iii) testing and referral, and iv) prevention (nutrition, cleaning, remediation). All the materials were assessed by three researchers, EM, EP, and KG. The researchers read through each of the materials and identified themes that were consistent with information about lead poisoning. In the cases where there was discordance in the themes presented in any of the materials, the principal investigator reviewed the materials and made a final decision. One point was awarded for each subtopic mentioned in the materials for a maximum of 13 points; testing and referral were treated as a single topic in these analyses.

### Statistical analyses

Descriptive statistics were employed to describe the distribution of the readability levels; median and interquartile range were used to describe the data and displayed in Box and Whiskers plots. The Kruskal-Wallis H test was conducted to determine if the readability levels differed between the materials obtained from different states. Frequencies of materials produced at less than an 8th grade reading level and less than a 5th grade reading level were determined. A Wilcoxon rank sum test was used to examine the relationship between readability level and number of topics included in the resources. For these analyses, readability levels were dichotomized to >8th grade and < 8th grade.

## Results

**Fig. 1:**
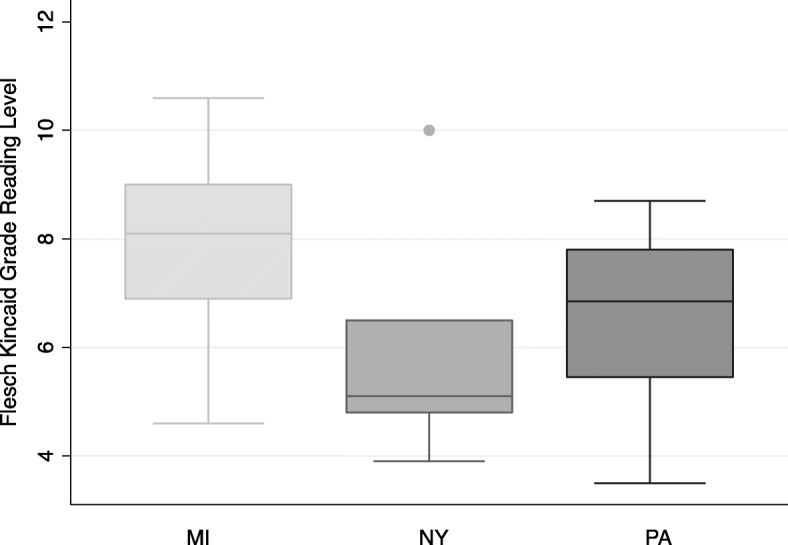
Flesch Kincaid Grade Level of informational materials from the three states

Readability level tests applied to 31 informational materials on lead and lead poisoning obtained from local and state health departments indicate a median readability level of 6.7 (IQR: 5.1–8.1). The readability level suggests that the resources can be read by individuals who have completed the 6th grade. However, there was considerable variability in the readability levels of the reading materials ranging from 3.5 to 10.6; some of the materials were prepared at a reading level appropriate for individuals who have completed the 3rd grade while other materials were suitable for individuals who have completed the 10th grade. Materials obtained from Michigan had the highest median reading level of 8.1 (IQR: 6.9–9.0) followed by Pennsylvania with a median reading level of 6.9 (IQR: 5.5–7.8). New York had the lowest reading level (median = 5.1; IQR = 4.8–6.5); Fig. 1 shows a descriptive summary of the readability levels from the three states.

Overall, 29% (*n* = 9) of the materials were produced at greater than an 8th grade reading level. Most of the materials prepared at >8th grade reading level were from Michigan (*n* = 6); followed by Pennsylvania (*n* = 2). Only one resource from New York was prepared at >8th grade reading level. Additionally, 23% (*n* = 7) of the materials were produced at <5th grade reading level; one resource from Michigan and three each from New York and Pennsylvania.

The median readability levels of the materials from the different states were significantly different from one another (Chi-squared statistic = 7.3 (df = 2), *p*-value = 0.03). Specifically, the readability of the materials from Michigan were significantly different from the readability levels of the materials from New York. The materials from Michigan had a higher median readability level (8.1) compared to the median readability of the materials from New York (5.1).

### Thematic content of materials

There was variation in the themes that the different materials addressed, in fact only one brochure, from PA, included all themes and subtopics. Only 14/31 (45%) of the materials addressed each of the main topics; definition of lead poisoning, risk factors and exposures, testing and referral, and prevention. Testing and referral was the theme that received the fewest mentions. On average, the NY materials mentioned 7 subtopics, and the PA materials mentioned 8 subtopics. The materials from Michigan mentioned between 1 and 3 subtopics-water as a source of lead poisoning, and/or one or two of the following three subtopics: the effects of lead poisoning, lead based paint as an exposure, and how to prevent lead poisoning.

With respect to old housing as a risk factor for lead poisoning, differences were observed between states; all the materials from PA mentioned old housing as a risk factor for lead poisoning compared to 60% of the materials from NY and 20% of the materials from MI. The materials from NY and PA focused on lead-based paint as a source of lead; at least 90% of the materials in these states mentioned lead-based paint as a source of exposure.

**Fig. 2:**
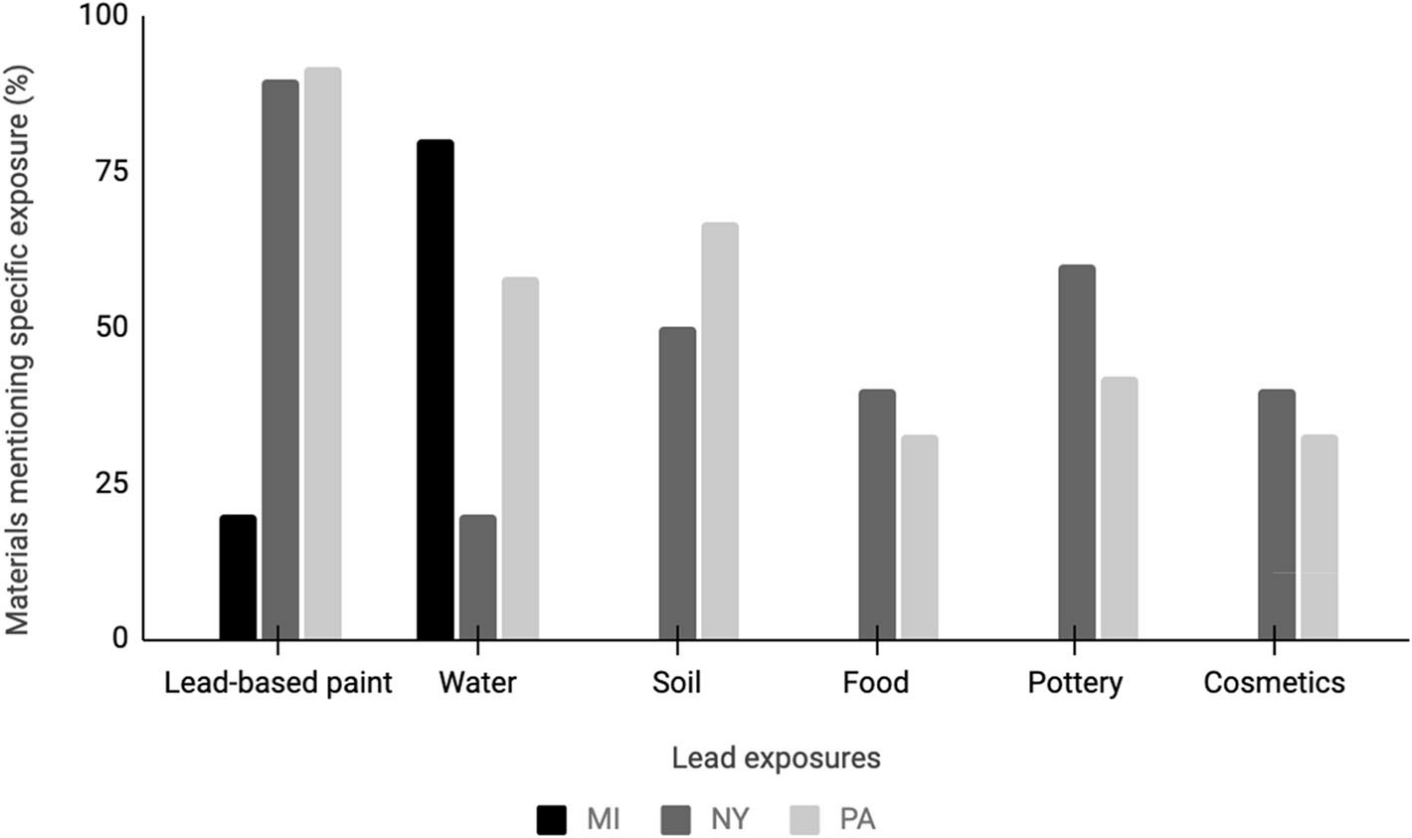
Proportion of the informational materials addressing each lead exposure

Most of the materials from MI (80%; *N* = 10) focused on water as a source of lead. The materials focused on how to prevent exposure to lead in water including as examples, letting the water run for at least 30 s before use, replacing faucet aerators, using cold water for cooking and drinking, and using a water filter. In comparison only 20 and 58% of the materials from NY and PA, respectively, mentioned water as a source of lead poisoning. Additionally, both these states addressed other sources of lead including soil, food, pottery and cosmetics; however, the materials from Michigan did not include any of these other sources of lead. Figure 2 shows a summary of the mention of lead exposures by state.

**Fig. 3:**
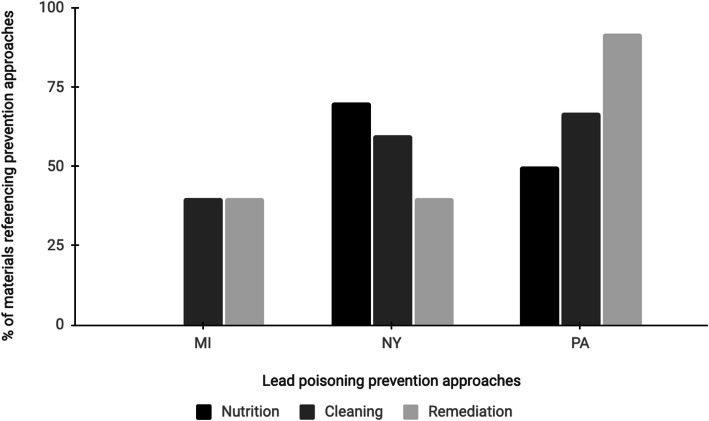
Proportion of the informational materials from each state addressing each of the lead poisoning prevention techniques

Likewise, as shown in Fig. 3, the emphasis on prevention of lead poisoning differed by state. The materials from New York and Pennsylvania refer to cleaning, nutrition and remediation as preventive techniques. However, the materials from Michigan only mention cleaning and remediation as preventive techniques and do not mention nutrition as a preventive technique.

Consensus on coding for thematic content was attained for 93% of the topics and subtopics. Most of the ambiguity arose in coding lead remediation as a prevention approach for the materials from MI. The ambiguity arose because lead remediation was implied in the materials but not explicitly stated.

The Wilcoxon rank sum test revealed that materials that were prepared at >8th grade reading level contained statistically significantly fewer topics than materials prepared at <8th grade reading level (Z = 2.55, *p* = 0.01). The median number of topics in the materials prepared at >8th grade reading level was 2 and the median number of topics in the materials prepared at <8th grade reading level was 8.5.

## Discussion

Contrary to our hypothesis, a majority of the materials were prepared at a 6th to 8th grade reading level as recommended by CDC and researchers [19, 21, 22]. Generally, informational materials aim to increase awareness of lead poisoning, and to inform, educate, and empower the audience on how to prevent, test for, and manage lead poisoning. Therefore, it is particularly important for the materials that may be accessed by the community independently of a provider (e.g on the internet, at community events/fairs) to be comprehensible by the intended audience. While several patient education materials have been assessed to have reading levels higher than the recommended reading levels, these lead poisoning materials were mostly compliant with the recommendations. This is probably an outcome of two factors: the language of lead poisoning does not contain complex medical terms while other health issues usually do [28], and lead poisoning has been an environmental issue for over a century and hence public health educators are intentional in preparing materials to ensure they are within the recommended reading levels.

An estimated 21% of the US population reads below a 5th grade level [29]. In our sample, one fifth of materials were prepared at <5th grade reading level appropriate for this population. While this is commendable, it is also concerning. Due to systemic racism and economic inequalities in the US, certain minority groups including Blacks and Hispanics are more likely to have access to fewer educational resources that might result in lower educational levels. The same population groups are at higher risk for lead poisoning. Therefore, the majority of the reading materials prepared at >5th grade reading level might not be appropriate for some groups that are high-risk for lead poisoning, including low-income, African American/Black families, and families of Hispanic ethnicity. Previous studies have highlighted racial differences in lead poisoning prevalence and risk factors; low income and/or African American children have higher blood lead levels [30, 31]. Public health practitioners need to consider educational levels and prepare materials that suit the readability needs of the population, and to disseminate the materials effectively. We recommend that materials on lead poisoning awareness and prevention be prepared at a 5th grade reading level.

There was notable variation in the themes addressed in the informational materials. Materials from New York and Pennsylvania included several sources of lead while materials from Michigan focused on water as a source of lead. This focus of water as a lead exposure in the materials from Michigan is likely informed by the Flint, Michigan water crisis and therefore contextually relevant for that state [24]. While materials from Michigan relevantly addressed water as the primary source of lead poisoning, they were almost silent on other lead exposures. Nationwide more than 70% of lead poisoning cases are attributed to lead based paint [32] and given that 64.6% of homes in Michigan were built before 1980 - compared to 77.2% in NY and 69.6% [33] - materials on lead poisoning would be more impactful if they included both water and lead based paint as potential sources of lead. Including multiple sources of lead in informational materials can increase awareness of the multiple exposures, especially for transient families that might live in a locale where water is the primary source of lead and then move somewhere with lead dust as the primary lead exposure.

Additionally, for some migrant families who might consume unregulated imported foods that might contain lead, information on foods as a potential source of lead might be beneficial. Studies from the CDC have identified spices and some herbal remedies as a possible source of lead exposure [34, 35] and have described an association between lead poisoning and ayurvedic medications in 5 states [36]. Other sources of lead that have resulted in elevated blood lead level cases in children include ceramics, and jewelry [37–39]. This suggests that public health practitioners should consider cultural make-up of the population, and content-specific lead exposures when preparing informational materials. Materials that are prepared at the recommended reading levels but not comprehensive with respect to the sources of lead are likely not to meet the needs of specific groups whose lead exposures might typically be considered less prevalent.

The mission of an organization preparing lead informational material can inform the content of the materials the same way that contextual situations can. For example, it is logical to assume that an organization like Women, Infants and Children would focus their messaging on nutrition as a lead poisoning prevention approach to align with their goal to help mothers and children up to age 5 learn about nutrition, make healthy food choices, and improve overall health. Public health practitioners need to consider what is the more efficacious approach in material preparation; guidance by contextual events and/or the goals of an organization, or developing a standard list of themes to be included in all published materials. There are pros and cons to each option. For example, in the Flint water crisis, while it was probably beneficial for the audience to learn that lead exposure can occur simultaneously from various sources, it was more salient for the audience to learn about, and focus on the most relevant exposure. However, a one-size fits all approach is likely to miss some high-risk groups.

### Limitations

This is a cross sectional study of a limited number of materials and hence temporal changes in readability levels and contents of the materials cannot be addressed. The time of publication of some of the materials might impact the topics mentioned in the materials and hence important to consider for comparisons purposes. However, these readability levels suggest that materials on lead poisoning prevention, currently available and in use, are prepared at appropriate reading levels. Nevertheless, the authors acknowledge that literacy levels contribute only partly to the readability; this study did not assess the totality of the materials including the layout, graphics, typography and learning stimulation. In addition, this study only assessed materials published online and a few hardcopy print materials from one location. It is likely that other organizations such as healthcare facilities print their own material that may or may not be at the recommended reading levels hence the results are not generalizable. Finally, to describe the relationship between FKGL score and number of topics in the materials, the FKGL score was dichotomized which may have resulted in loss of information. However, dichotomizing the FKGL score allows for meaningful interpretation and application on the part of public health practitioners.

## Conclusion

Lead poisoning and lead poisoning prevention materials aimed at increasing awareness on lead poisoning, lead poisoning exposures and promoting behavioral practices for prevention are mostly prepared at the recommended reading levels. However, populations at highest risk of lead poisoning might benefit from materials prepared at even lower reading levels. Heterogeneity existed in the main themes that were covered and the number of subtopics addresses; although all the materials contained contextually relevant materials, omission of some of information, such as less prevalent sources of lead poisoning, can leave some at-risk groups vulnerable to lead poisoning. We suggest increasing population access to appropriately prepared materials with respect to readability level and content might facilitate increased awareness about lead poisoning, especially in high-risk populations, which may lead and contribute to lowering the incidence of lead poisoning in children under the age of 6 years.

## Data Availability

The datasets used and/or analyzed during the current study are available from the corresponding author on reasonable request.

## Abbreviations

PEM: Patient Education Materials
CDC: Centers for Disease Control and Prevention
PA: Pennsylvania
NY: New York
MI: Michigan
FKGL: Flesch Kincaid Grade Level
